# A Longitudinal Analysis of Regional Brain Volumes in Macaques Exposed to X-Irradiation in Early Gestation

**DOI:** 10.1371/journal.pone.0043109

**Published:** 2012-08-14

**Authors:** Kristina Aldridge, Lei Wang, Michael P. Harms, Amanda J. Moffitt, Kimberly K. Cole, John G. Csernansky, Lynn D. Selemon

**Affiliations:** 1 Department of Pathology and Anatomical Sciences, University of Missouri School of Medicine, Columbia, Missouri, United States of America; 2 Departments of Psychiatry and Behavioral Sciences and Radiology, Northwestern University Feinberg School of Medicine, Chicago, Illinois, United States of America; 3 Department of Psychiatry, Washington University School of Medicine, St. Louis, Missouri, United States of America; 4 Department of Neurobiology, Yale University School of Medicine, New Haven, Connecticut, United States of America; King’s College London, United Kingdom

## Abstract

**Background:**

Early gestation represents a period of vulnerability to environmental insult that has been associated with adult psychiatric disease. However, little is known about how prenatal perturbation translates into adult brain dysfunction. Here, we use a longitudinal study design to examine the effects of disruption of early gestational neurogenesis on brain volume in the non-human primate.

**Methods and Principal Findings:**

Five Rhesus macaques were exposed to x-irradiation in early gestation (E30–E41), and four control monkeys were sham-irradiated at comparable ages. Whole brain magnetic resonance imaging was performed at 6 months, 12 months, and 3 and 5 years of age. Volumes of whole cerebrum, cortical gray matter, caudate, putamen, and thalamus were estimated using semi-automated segmentation methods and high dimensional brain mapping. Volume reductions spanning all ages were observed in irradiated monkeys in the putamen (15–24%, p = 0.01) and in cortical gray matter (6–15%, p = 0.01). Upon covarying for whole cerebral volume, group differences were reduced to trend levels (putamen: p = 0.07; cortical gray matter: p = 0.08). No group-by-age effects were significant.

**Conclusions:**

Due to the small number of observations, the conclusions drawn from this study must be viewed as tentative. Early gestational irradiation may result in non-uniform reduction of gray matter, mainly affecting the putamen and cerebral cortex. This may be relevant to understanding how early prenatal environmental insult could lead to brain morphological differences in neurodevelopmental diseases.

## Introduction

Early gestation has been identified as a particularly vulnerable period for human brain development. For example, human populations exposed to atomic radiation from the World War II bombings or to radiation emissions from the Cherynobyl nuclear plant who were age 8–25 weeks of gestation exhibit brain dysfunction, with the most severe damage occurring at 8–15 weeks for the atomic irradiation [1], [2]. Substance abuse in early gestation is also detrimental to normal brain development as heavy maternal alcohol consumption in the first trimester has been linked to anxiety and depression disorders in offspring [3]. In addition, prenatal nicotine abuse has been identified as a risk factor for Conduct Disorder, and though less well established, there is also some evidence to support an association between prenatal smoking and attention deficit hyperactivity disorder (ADHD) [4], [5]. Moreover, a wide range of prenatal environmental stressors, e.g., maternal infection, maternal malnutrition, Rh incompatibility, have been shown to increase the incidence of adult schizophrenia, and the first two trimesters of human fetal development have been identified as periods of particular susceptibility [6]–[13]. Recent findings also suggest that maternal immune reactivity to the fetus may account for at least a subset of autistic spectrum disorders [14].

In the present study we disrupted neurogenesis during early gestation in the macaque brain by exposure to x-irradiation. Although irradiation kills dividing neurons indiscriminately, temporal staggering of neurogenesis in the macaque brain allows for specificity in targeting different brain regions via differential timing of the radiation exposure. For example, the thalamus is generated early in gestation (E36–E45) [15], [16], and neurogenesis in the striatum, although more prolonged, overlaps this same early developmental period (E36–E80) [17]. In contrast, corticogenesis begins slightly later and extends throughout midgestation (E45–E102) [18], [19]. Thus, we hypothesized that exposure to radiation in early gestation would result in widespread subcortical deficits even at the earliest ages examined because the radiation would be lethal to newly born neurons in these structures [15]–[17]. Conversely, we postulated that the cortex would not exhibit a volumetric deficit in infant monkeys because the irradiation occurred before the onset of corticogenesis; rather, volume reduction might emerge during postnatal ontogeny in conjunction with postnatal maturation of connectivity and synaptic pruning [20].

The present study addresses the following questions: 1) Are brain volume reductions observed following early gestational exposure to radiation? 2) Are these volume reductions confined to subcortical structures in which newly generated neurons are killed by the radiation?, and 3) Are volume reductions present in infancy, or do they emerge at later maturational ages?

## Materials and Methods

### Subjects

Nine Rhesus macaque monkeys were exposed to radiation (three males/two females) or were sham irradiated (two males/two females) *in utero* (Table 1) and then underwent repeated, structural MRIs at four postnatal time points. All animals in this study were housed, fed, experimentally treated and imaged in accordance with protocols approved by the Yale Institutional Animal Care and Use Committee.

**Table 1:** Time and Dose of Irradiation Exposure.

Animal	Sex	DOB	Time in embryonic day (E) and dose of irradiation (in cGy)	Total dose (cGy)
**Irradiated subjects**				
FIM-Tm	M	021901	E34(50), E36(50), E38(50), E41(50)	200
FIM-Lf	F	032301	E30(50), E33(50), E35(50), E37(50)	200
FIM-Sf	F	042701	E32(50), E34(50), E36(50)	150
FIM-Bm	M	052601	E35(50), E37(50), E38(50)	150
FIM-Am	M	083002	E33(50), E35(50), E37(50)	150
**Control subjects**				
CON-Mm	M	032301	4 doses ketamine- E29, E32, E34, E36	0
CON-Om	M	042501	2 doses ketamine- E34, E36	0
CON-Vf	F	073101	3 doses ketamine- E39, E41, E43	0
CON-Lf	F	072902	4 doses ketamine- E32, E34, E37, E39	0

DOB = date of birth; cGy = centigray.

### Fetal Irradiation

*In utero* exposure to x-rays was performed as described in detail by Algan and Rakic [21] and is only briefly described here. Pregnant monkeys were sedated with ketamine (5–10 mg/kg), administered in conjunction with atropine (0.02 mg/kg). The head of the fetus was located using ultrasound in order to estimate the depth of the head from the abdominal surface. A 250 kV, 15 mA Stabilipan x-ray tube delivered a beam of irradiation (area 5×5 cm) to the head of the fetus. The duration of the exposure required for the desired dose of irradiation was calculated based on the depth of the head in the abdominal cavity. The fetuses were exposed to x-rays in early gestation (E30–E41). Multiple exposures (3–4) of 50 cGy were delivered usually every other day for a total exposure dose of 150–200 cGy (Table 1). Sham irradiation consisted of the same protocol used for irradiation, e.g., sedation with ketamine and ultrasound localization, but minus exposure to the x-ray beam.

Following irradiation or sham irradiation, the status of pregnant monkeys was carefully monitored and viability of the fetus was confirmed by ultrasound throughout the remainder of the gestational period. All monkeys in this study, with the exception of CON-Lf and FIM-Am, were born naturally at term (∼E165) and allowed to stay with their mothers until 6 months of age. CON-Lf had to be delivered via cesarean section because the pregnancy went several days beyond term. FIM-Am, although delivered naturally, was rejected by his mother. Both of these animals therefore spent the first 6 months in the primate nursery in the Yale University School of Medicine. At 6 months of age, the monkeys were pair-housed (n = 7) or singly-housed (CON-Vf and FIM-Sf) in the adult non-human primate colony at Yale. Mean age and body weight did not differ significantly between groups at any of the four postnatal time points sampled (Table 2).

**Table 2:** Age and Body Weight at Scanning.

Animal	6 months		12 months		3 years		5–6 years	
	Age	Weight	Age	Weight	Age	Weight	Age	Weight
**Irradiated subjects**								
FIM-Tm	0.58	1.4	1.0	2.4	3.08	4.5	5.92	8.0
FIM-Lf	0.50	1.4	1.0	2.1	3.17	5.0	6.0	7.0
FIM-Sf	0.50	1.5	1.0	1.8	3.17	6.5	5.96	7.5
FIM-Bm	0.50	1.8	1.0	2.5	3.17	7.0	5.71	10.5
FIM-Am	0.58	1.4	1.08	2.0	3.21	4.6	5.08	9.5
**Control subjects**								
CON-Mm	0.50	1.8	1.0	2.6	3.17	6.3	5.88	9.5
CON-Om	0.50	1.9	1.0	2.4	3.17	5.5	5.92	10.0
CON-Vf	0.58	1.7	1.0	2.5	3.04	5.5	5.54	7.0
CON-Lf	0.58	1.8	1.16	2.6	3.33	7.6	5.13	9.0

Weight in kg; Age in years.

### Structural Magnetic Resonance Imaging

High-resolution 3D T1-weighted MR scans were collected at 6 months, 12 months, 3 years and 5–6 years. Due to the length of this study (>5 years), imaging was performed on three different scanner platforms. Within a given age group, animals were generally scanned on the same platform, except for one FIM (Am) and one CON (Lf) monkey who were approximately one year younger than the others. Scans for these two monkeys were performed one year later than same age scans of the other monkeys, and therefore scans at 12 months and 3 years of age were acquired on a different platform from the other monkeys at the same age. Specifically, the 6 month MR scans of all animals and the 12 month scans of all but those two animals (FIM-Am and CON-Lf) were acquired on a 1.5T LX GE Advantage scanner using a SPGR sequence. The 12 month scans of FIM-Am and CON-Lf, and the 3 year scans of all other animals were collected on a 1.5T Siemen’s Sonata scanner, using a 3D FLASH acquisition. The 3 year scans of FIM-Am and CON-Lf and the 5–6 year scans of all animals, including FIM-Am and CON-Lf, were acquired on a 3T Siemen’s Trio scanner using a 3D MPRAGE acquisition. Resolution was 0.63×0.63×0.7 mm for the scans at 1.5T and 0.63×0.63×0.6 mm for the scans at 3T (except for the 3 year scans of FIM-Am and CON-Lf, who were inadvertently scanned at a resolution of 0.5×0.5×0.5 mm). When multiple T1-weighted scans were collected for a monkey at a given age, those volumes were registered and averaged prior to further analysis.

### Measurement and Segmentation of Whole Cerebral Volume and Cortical Gray Matter

Whole cerebral volumes (WCV) containing cerebral cortical gray matter (CGM) and white matter, subcortical gray matter, and diencephalon were manually segmented from the MR image of the entire cranium using Analyze 8.0 software [22]. Voxels containing cerebrospinal fluid, brain stem structures, and cerebellum were excluded. Manual segmentations of WCV and CGM were performed by two of the authors over a two year period (AJM and KKC), following acquisition of the 3-year scans. Volumes of WCV and CGM were calculated by summing the number of voxels enclosed within these segmentations and then multiplying the sums by the voxel dimensions. Inter-rater reliability was assessed as the percent volume difference in CGM between raters (blind to condition) in three randomly selected scans, following Collins et al [23]. The average percent volume difference was 9.7%. The volume difference attributed to inter-rater variance is in the same range (∼10%) reported for comparisons among automated registration algorithms [24].

### High Dimensional Brain Mapping and Measurement of Subcortical Gray Matter Volumes

**Figure 1:**
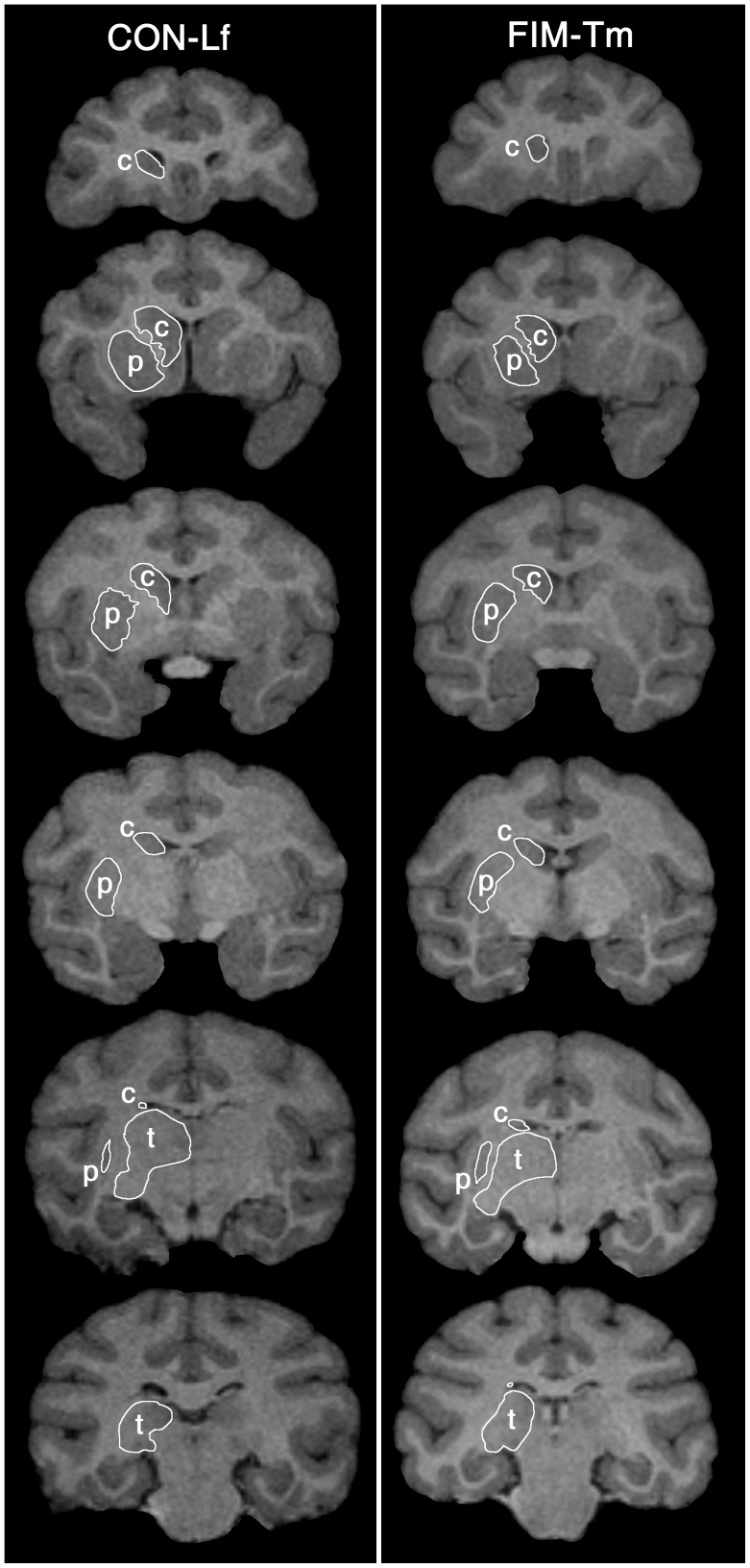
Illustration of example surfaces of subcortical structures. The right thalamus (t), caudate (c), and putamen (p) are outlined in analogous slices spanning the rostrocaudal extent of the structures in one CON and one FIM at 5 years (radiological convention shows the subject’s right side on the left side of the image).

A semi-automated procedure was used to measure volume of the thalamus, caudate, and putamen by a single author (KA). First, an MRI scan collected from a control Rhesus macaque, not included in this study, was used to construct a neuroanatomical template for the each of the three structures of interest. The neuroanatomical template was created by manually outlining the caudate nucleus, putamen, and thalamus in the template MR scan. The neuroanatomical boundaries of these structures were identified with the aid of an atlas [25] and by consultation with an expert in nonhuman primate neuroanatomy (Dr. Joseph Price, Washington University School of Medicine). This template scan and each scan included in the study were landmarked at pre-defined positions within the striatal-thalamus complex. Next, high dimensional brain mapping (HDBM) was then applied to these data in a two-step procedure as described previously [26]. In the first step, the template scan was coarsely aligned to each target MR scan using the landmarks. Next, a probabilistic transformation of the template voxels was performed [26], [27]. This procedure produced a segmentation of each structure in each target scan (Figure 1). Each of the segmentations produced by HDBM was then inspected and any errors in anatomical boundaries introduced during the HDBM registration process were manually corrected, if necessary. Volumes of the structures were calculated by multiplying the number of voxels enclosed within these final segmentations by the voxel dimensions. Volume measures for left and right were summed for each structure to reduce the number of statistical comparisons performed.

Intra-rater reliability for the single rater for these data was assessed in two ways. First, following Kohn & Cheverud [28] and Aldridge et al. [29] the three volume measures are used as dependent variables in separate, nested analyses of variance (ANOVA) with two effect terms: 1) subject and 2) trial nested within subject. The proportion of the total variance due to the different subjects represents the between-subject variance. The proportion of the total variance due to differences between the two trials of the same subject represents the within-subject variance, or intra-rater error. The proportion of the total variance due to intra-rater error is 0.1% for the thalamus, 2.3% for the caudate, and 7.3% for the putamen, indicating that a very small proportion of total variance is due to the rater. Second, intra-rater reliability was also assessed as percent volume difference between repeated mapping and manual correction trials in three randomly selected scans [23], with the following results: thalamus: <1%; caudate: 4.1%; putamen: 8.9%. These findings are comparable to that of previous studies of intra-rater error in manual segmentation of the caudate and putamen, including Collins et al [23].

### Statistical Analysis

Structural volumes were analyzed in a repeated measures mixed model (PROC MIXED in SAS 9.2, SAS Institute Inc., Cary, NC). Age was treated as a repeated measure class variable (rather than a continuous variable) to avoid assumptions about the trajectory of the changes over time, as would be required for random effect growth-curve modeling. Covariance of the model residuals across ages was modeled with a Huynh-Feldt covariance structure that was assumed to be the same for the FIM and CON groups. The Huynh-Feldt covariance structure (“type = hf" in PROC MIXED) allowed the estimation of separate variances at each age, with the covariance between ages constructed from the arithmetic means of these variances. Degrees of freedom were calculated using the method of Kenwood and Roger [30]. Analyses were conducted both with and without WCV included as a covariate (except for the analysis of WCV itself). By using PROC MIXED for the analysis, rather than PROC GLM, this covariate was allowed to vary within a subject, consistent with the fact that WCV was measured separately at each age. To preserve degrees of freedom, the models with WCV as a covariate assumed a single linear relationship that applied across all ages and for both groups (i.e., we did not attempt to model age-by-WCV or group-by-WCV interaction terms). Initial analyses included group, age, and group-by-age as fixed effects. However, the group-by-age interaction was not significant for any structure (p = 0.13 for WCV; p>0.23 for all other structures with or without WCV as a covariate), and thus to preserve degrees of freedom in testing for a main effect of group, the analyses presented henceforth included only the main effects of group and age as modeled factors. Within this reduced model, the effect of age was significant in all analyses (p<0.02), except for the putamen (p = 0.67 and 0.43 without and with WCV as a covariate, respectively).

To investigate the possible impact of changes in scanner platform on our results, we examined models with group, age, and scanner as fixed effects. Inclusion of scanner platform had no appreciable impact on the p-values for the effect of group for any of the structures (relative to the model with just group and age as effects), and while the significance of the effect of age was generally reduced, it remained significant for the whole brain, cortical gray matter, and caudate (p<0.03). Notably, the effect of scanner itself was non-significant for all structures (p>0.15).

## Results

**Figure 2:**
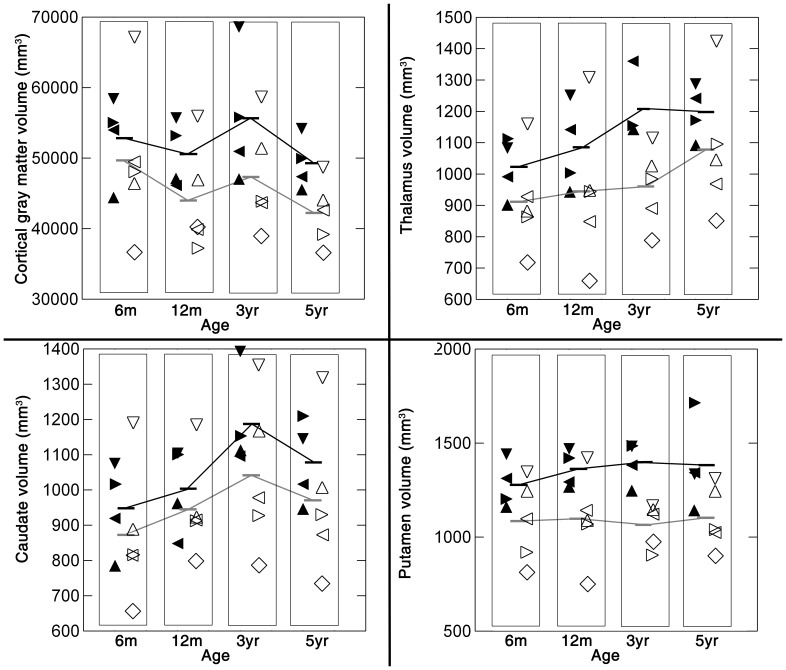
Volume measures plotted by age. Subjects in the CON group are represented by solid symbols, while the FIMs are illustrated with open symbols. The symbols for each individual are: ▾ CON-Mm, ▸ CON-Lf, ◂ CON-Om, ▴ CON-Vf, ▿ FIM-Bm, ◃ FIM-Tm, ▹ FIM-Sf, ▵ FIM-Lf, ⋄ FIM-Am. Mean group values are illustrated by a bar and connected by lines across time points for each group (CONs in black, FIMs in gray).

Raw volume data are plotted in Figure 2. Despite differences in group means for each of the structures measured at each time-point, there was some overlap in the distributions of the groups. The distributions of the data for FIMs (open symbols) were broader than those of the CONs (solid symbols).

### Whole Cerebral Volume

WCV did not differ significantly between groups (p = 0.27; Table 3).

**Table 3:** Mean volumes and percent mean reductions between CON and FIM for each structure.

		6months	1year	3years	5–6years
**Whole cerebral** **volume**	CON	85602	82663	91710	89651
	FIM	76244	74734	78138	78047
	% difference	10.9%	9.6%	14.8%	12.9%
**Cortical gray** **matter**	CON	52914	50519	55620	49234
	FIM	49576	43979	47305	42212
	% difference	6.0%	12.9%	14.9%	14.3%
**Thalamus**	CON	1022	1085	1206	1197
	FIM	910	944	979	1077
	% difference	10.9%	13.0%	20.4%	10.0%
**Caudate nucleus**	CON	949	1004	1188	1079
	FIM	873	945	1043	972
	% difference	8.0%	5.8%	12.2%	10.0%
**Putamen**	CON	1277	1362	1398	1382
	FIM	1084	1096	1065	1103
	% difference	15.1%	19.5%	23.8%	20.2%

### Cortical Gray Matter Volume

CGM was significantly smaller in FIMs compared to CONs (p = 0.01). However, when WCV was included as a covariate, the difference was reduced to trend levels (p = 0.08). CGM was reduced in FIMs approximately 6% at 6 months, 13% at 1 year, 15% at 3 years, and 14% at 5 years.

### Thalamus Volume

Thalamic volume exhibited trend reductions in FIMs at all ages (11% at 6 months, 13% at 1 year, 20% at 3 years, and 10% at 5 years). The difference between groups did not reach statistical significance without covarying for WCV (p = 0.09); inclusion of WCV as a covariate nullified this trend (p = 0.73).

### Caudate Nucleus Volume

Caudate volume did not differ significantly between groups (p = 0.82 without covarying for WCV; p = 0.24 covarying for WCV).

### Putamen Volume

Putamen volume was reduced in FIMs on average at all four ages (15% at 6 months, 20% at 1 year, 24% at 3 years, and 20% at 5 years). This reduction was statistically significant without WCV included as a covariate (p = 0.01) and reduced to trend levels with WCV as a covariate (p = 0.07).

## Discussion

Macaques exposed to radiation in early gestation appear to exhibit non-uniform reductions in gray matter volume, observed most prominently in the putamen and cerebral cortex. Volume differences were present at the youngest age (6 months) examined even in the cerebral cortex. The presence of cortical reductions following early gestational disruption of neurogenesis suggests vulnerability of brain circuits to early gestational environmental insult.

### Technical Considerations

This study is based on a relatively small number of subjects: 5 FIM and 4 CON. The lengthy experimental paradigm, spanning 6 years and involving prenatal irradiation and repeated postnatal MRI scans, limited the numbers of subjects in this study. Although mean volumes were consistently lower in FIMs across all brain structures, including whole cerebral volume, significant differences between groups were not always found. The lack of significance may in part have been due to what appears to be a higher degree of variability in brain volume in the irradiated group compared to controls. Thus, with a larger number of subjects, additional significant findings might have been observed.

It should be emphasized that the FIMs examined in the present study were healthy animals. Their mean body weight did not differ from that of CONs. One FIM had to be raised in the Yale primate nursery after being rejected by his mother, as was one CON who had been delivered by Caesarian section and not able to stay with her mother. Both were hand fed in the nursery and thrived. In fact, FIMs were virtually indistinguishable from CONs throughout their postnatal maturation. They exhibited normal home cage behavior, including feeding and drinking. The only abnormalities detected in FIMs were circling behavior and restricted cognitive deficits, the latter emerging only in adulthood [31].

Imaging was performed on three different scanner platforms over the 6 year course of this study, due to a combination of institutional decisions (necessitating the switch to the Siemen’s Sonata) and a desire to collect the highest quality anatomical images (the switch to the Siemen’s Trio). The changes in scanner platform and imaging sequence may have introduced some bias and increased the overall variance of the volume measurements [32], [33]. However, this effect is likely to be quite small relative to the natural volumetric variability across individuals [34], [35], meaning that the power of our study to detect changes was most likely limited by sample size, rather than a (subtle) increase in variance due to changing scanner platform. Further, previous studies have shown that the effects of disease on measurements of brain structure are substantially greater than the effects of differences in scanner platforms [36], [37]. Critically, the imaging of FIM and CON monkeys remained balanced across scanner platform and thus both the group comparisons and our original tests for group-by-age effects (see Methods) should be relatively insensitive to the scanner changes unless there was an interaction between scanner platform and group, which seems unlikely [37].

We have analyzed brain volumes with and without covarying for whole cerebral volume. Significant group differences were not found.when volumes were covaried for whole cerebral volume. Although covarying for brain volume has become standard procedure for taking account of individual variability in brain size in neuroimaging studies of human disease, correcting for whole brain volume when studying developmental disorders, or in this instance the impact of prenatal irradiation, that lead to smaller overall cerebral volumes prevents detection of the actual pathology since all individuals in the experimental group would be expected to have smaller brain volumes than the controls [38], [39].

### Subcortical Volume Reductions in Fetally Irradiated Monkeys

Reduction of gray matter volume in the putamen may be due to the direct lethal effect of radiation on newly born neurons in the putamen [17]. Radiation administered when neuronal progenitor cells are undergoing their final mitotic division to produce neurons destined for a particular structure will impact neuron number in that structure more severely than in other brain regions that either have not begun or have already finished generating neurons. Interestingly, the putamen appears to have been impacted more by the early gestational irradiation than the caudate nucleus, which did not show significant group differences. The differential vulnerability of these two structures may reflect the fact that large neurons are generated a few days earlier in putamen than in the caudate and therefore the timing of the radiation exposure may have coincided more closely with the period of neurogenesis for large cells in the putamen [17]. Alternatively, failure to detect reductions in thalamic and caudate volumes in the present study might simply be accounted for by the rather large individual variability in the irradiated group, which included the animals with largest and smallest thalamic and caudate volumes. In this regard, it was somewhat surprising that significant group differences were not observed in the thalamus. Previously, we have found reductions in adult thalamic volume following exposure to x-irradiation in early gestation [26], [27].

### Cortical Volume Reductions in Fetally Irradiated Monkeys

We hypothesized that the cortex might be spared at early postnatal ages since the early gestational irradiation would not have directly killed newly generated cortical cells [18], [19]. Instead, reduced cortical volumes seem to have been present from infancy in the irradiated monkeys. Although the mean deficit observed at the earliest age examined (6% at 6 months) was smaller than that observed (13%) at one year, this apparent increase in the cortical gray matter reduction over time was not sufficiently strong to be confirmed statistically. The reduction of cortical volume following early gestational exposure to x-irradiation may be due to irradiation-induced killing of cortical progenitor cells as these early dividing cells serve to expand cortical surface area [40]–[42]. A recent study of morphometric alterations in prefrontal area 46 in adult animals, including the subjects in this study, support this premise as prefrontal cortical surface and volume were diminished while cortical thickness was unchanged [Selemon et al., in review]. Another possibility is that cortical volume reductions are consequent to decreased afferent input, in particular from the thalamus. This could occur as the result of loss of neuropil contributing to the reduction of volume or perhaps to developmental regulatory mechanisms that match thalamic neuron number to cortical size [43]–[45] albeit the lack of a significant thalamic volume reduction argues against this possibility. The potential reductions in cortical gray matter volume in FIMs in this study, and in a prior cohort of monkeys exposed to irradiation in early gestation [27], suggest that cortical volume may be reduced by a prenatal insult occurring even before cortical neurons are generated. In accord with this supposition, we previously reported that FIMs, specifically the cohort examined in the present study, exhibited profound adult-onset cognitive deficits on a working memory task [31]. Our MRI results are in accordance with those of Short et al [46], who found that maternal influenza infection during gestation also resulted in significant reductions of cortical gray matter in the non-human primate brain, highlighting the potential for early gestational insult to negatively impact development of the cerebral cortex.

### Longitudinal Brain Growth Patterns in Comparison to Human MRI Studies

Volume plots for the control monkeys in this study may serve to illustrate the normative brain growth pattern for the macaque monkey. Plots for the prenatally irradiated monkeys generally paralleled curves for the sham-irradiated monkeys though volumes were smaller for the irradiated monkeys. To our knowledge, there has been only one other longitudinal analysis of brain volume in the non-human primate, and that study did not parcellate brain regions [47]. Therefore, given the rarity of our dataset, a few general observations are worth noting despite the small number of subjects. The growth curve of the putamen appears to be flatter in comparison to all other structures examined, i.e., thalamus, caudate and cerebral cortex. This potentially distinct growth curve for the putamen is analogous to what has been observed in a large cross-sectional analysis of human subjects ranging in age from 4 to 18 years [48]. In human subjects, cortical gray matter volume either exhibited a steady decline from childhood to adulthood [48] or showed increasing volume to a peak in adolescence and then decline to adulthood [49]. Thalamic volume increased over this same maturational period in the human subjects with a slight decline from 15–18 years [48]. In contrast to the flat growth curve found in the caudate of human subjects [49], we observed a pattern of peak and decline in caudate volume in the small number of macaques included in this study. While it is possible that the age-associated changes in macaque caudate volume are due to sampling error, all nine individuals exhibited the same pattern of growth and decline.

### Potential Relevance to Neuropsychiatric Disease

Our findings suggest that a relatively mild insult in early gestation may result in multifocal reductions of gray matter volume. These findings highlight the vulnerability of the developing brain in the early embryonic period. As such, these findings might be relevant to human psychiatric conditions, including schizophrenia, ADHD, and autism, in which mounting evidence suggests a link to adverse prenatal events.
